# Fatigue among children and adolescents with acquired brain injury in a specialized neurorehabilitation setting

**DOI:** 10.3389/fresc.2024.1454602

**Published:** 2024-11-21

**Authors:** Marie-Louise Smidt Proschowsky, Sofie Hur Reimers, Anette Granhøj

**Affiliations:** Virum Brain Injury Center (VBI)—Capital Region of Denmark, Copenhagen, Denmark

**Keywords:** fatigue, rehabilitation, children, adolescents, acquired brain injury

## Abstract

**Introduction:**

We investigated the fatigue experienced in children and adolescents with acquired brain injury (ABI) undergoing neurorehabilitation.

**Methods:**

Fatigue was assessed using the pediatric quality of life inventory™ (PedsQL™). Multidimensional Fatigue Scale in 38 participants aged 2–19 years with ABI. Data were collected at enrollment and discharge, either from the participants themselves or their parents. The causes of ABI, including stroke, infection, tumor, and traumatic brain injury), were compared.

**Results:**

Participant-reported fatigue levels significantly decreased over time (*p* = 0.005), whereas parent-reported fatigue levels did not show a significant change. Fatigue levels varied by ABI cause, with stroke-associated fatigue having the least impact and infection-related fatigue showing the greatest impact.

**Conclusion:**

This study highlights the importance of individualized assessments that consider varying etiological factors and advocates for tailored interventions. Further research is needed to fully understand the long-term impacts of fatigue in this population.

## 1 Introduction

Acquired brain injury (ABI) refers to any injury to the brain that occurs after birth, either due to a traumatic event or an internal disease process that leads to damage in brain tissue (1). In children and adolescents, ABI occurs at a particularly vulnerable time because the brain is still developing (2, 3). The literature documents a wide range of typical sequelae, including intellectual, executive, physical, and language deficits that persist over time and frequently impact the quality of life (4–9). Similarly, studies report that fatigue is one of the most commonly reported symptoms, significantly affecting daily functioning (10–12). While there is no consensus on how to define fatigue (13), it is generally understood as a multidimensional concept that encompasses physical, mental, and emotional components (10, 12, 14–17). In clinical settings, fatigue is often defined as difficulty in initiating or sustaining voluntary activities (18).

A review by Wilkinson (19) found that fatigue affects the majority of children and adolescents with ABI, and it is associated with poor academic performance, reduced physical activity, and emotional difficulties. Fatigue has also been observed to persist for years after the onset of childhood and adolescent ABI (7, 20–22). Meeske et al. (23) reported that children with ABI caused by brain tumors (ages 2–18) experience significant fatigue for years, and this fatigue is linked to a lower health-related quality of life (HRQOL). Given that mental fatigue is associated with poor long-term outcomes, there is a critical need to address and assess it in clinical settings.

In adults with ABI, fatigue is also one of the most common and persistent sequelae (24–26), and it is well-documented with various instruments available for its measurement and monitoring (27, 28). However, brain injury in children occurs during a time of ongoing developmental processes, meaning that injuries at this stage can have particularly severe consequences. Some aspects of cognitive development are critically dependent on specific cerebral structures and networks that develop at certain stages (29). Therefore, findings from adult ABI research cannot be directly applied to pediatric cases. Despite its importance, there is limited research on fatigue in children and adolescents with ABI, although interest in this topic is growing. Only a few assessment scales cover the age range from early childhood to young adulthood. To our knowledge, the only internationally recognized outcome measure for fatigue in this age group (0–30 years) is the Pediatric Quality of Life Inventory Multidimensional Fatigue Scale (PedsQL MFS) (30). This scale has been translated into multiple languages and has been used in several studies on pediatric ABI (10, 11, 21, 31).

Research is typically based on patient follow-ups months or years after treatment in hospitals or rehabilitation centers. In many of these outpatient rehabilitation studies, children and adolescents with mild traumatic brain injury (mild TBI) are highly overrepresented (11, 14, 32). This overrepresentation can be problematic, as the mild TBI group is poorly defined and differs significantly from groups with moderate to severe ABI, who have verified irreversible lesions to brain tissue (33).

Little is known about children and adolescents with moderate to severe ABI during interdisciplinary neurorehabilitation after hospital discharge. According to most studies, fatigue is a disabling symptom that affects nearly all children and adolescents in rehabilitation to some degree. However, practitioners need more knowledge about how children and adolescents experience fatigue over time in a sub-acute rehabilitation setting and how fatigue differs according to the cause of injury. In this study, the PedsQL MFS was administered at the beginning and end of rehabilitation to gather information on fatigue symptoms and factors that may influence these symptoms. The main objectives were to describe: (1) the progression of fatigue during rehabilitation, including potential differences between child and parent ratings, and (2) the impact of different injury types on fatigue symptoms, with the overall goal of obtaining information to guide clinical practice and provide foundational knowledge for future studies.

## 2 Materials and methods

### 2.1 Participants

In the present study, the sample consisted of 38 children and adolescents, along with their parents. The inclusion criteria required enrollment in a specialized intensive rehabilitation program, a diagnosis of ABI, and an age range of 2–19 years. The study initially included all children and adolescents registered at the Virum Brain Injury Center (VBI) during the study period, regardless of time since injury, type of injury, or age. The majority of VBI patients are children and adolescents with moderate-to-severe ABI between the ages of 0–25 years. The definition of moderate-to-severe ABI in this context refers to patients with significant visible damage on MRI scans, neurological deficits, and such considerable consequences following the injury that they require prolonged, intensive, and interdisciplinary rehabilitation after hospital discharge. Additionally, children and adolescents with traumatic brain injuries meet the criteria for moderate to severe brain injury. Moderate head injuries are characterized by a GCS (Glasgow Coma Scale) score between 9 and 13 (in children aged over 5 years), a loss of consciousness for >5 min, and/or post-traumatic amnesia lasting 1–24 h, along with any potential neurological deficits. Severe head injuries are identified by a GCS score of 8 or below and/or post-traumatic amnesia lasting >25 h (34, 35).

Initially, the cohort included 45 children. However, reports from seven participants and/or their parents were later excluded from the analysis. Some were excluded because the cause of injury did not align with the conceptual definition of ABI, such as cases involving neurodegenerative disorders (1). Other patients were excluded due to incomplete consent forms.

### 2.2 Setting and intervention

VBI is a specialized rehabilitation center that treats children and adolescents with ABI who require interdisciplinary neurorehabilitation from hospital discharge until their reintegration into everyday life in their local communities (e.g., preschool institutions, schools, or youth education) (36). The rehabilitation at VBI is tailored to the individual needs and motivations of each child or adolescent, though many components are consistent across the multidisciplinary rehabilitation programs. All participants were engaged in outpatient rehabilitation (Monday to Friday, daytime) while living at home with their families. Each participant was assigned to a specialized team consisting of a neuropsychologist, a speech-language therapist, a physiotherapist, an occupational therapist, a special needs teacher, and a neuro-educator (pedagogue). The team was responsible for planning and delivering an individualized rehabilitation program, five hours a day, five days a week. In cases where fatigue was present, all families received psychoeducation about mental fatigue following ABI, along with consultations throughout the rehabilitation program. If the children were old enough, they participated in consultations with their neuropsychologist either individually or in small groups. Adolescents were trained in fatigue management and “brain breaks” with their occupational therapist. All children and adolescents received physical training with their physiotherapist multiple times each week.

### 2.3 Collection of data

The questionnaires were distributed by occupational therapists in paper form during the first month of the children/adolescents’ rehabilitation program at the VBI Center. The questionnaires were completed by the children, adolescents, and/or parents either at home or at the VBI Center. Due to young age or specific disabilities, some of the children and adolescents were unable to participate independently, and some were verbally assisted by the occupational therapist, who clarified terms and concepts. The reports were collected as part of routine assessments in the multidisciplinary rehabilitation program. Data collection occurred from September 2020 to January 2023.

The type of injury, date of injury (if available), and time since injury were identified from patient records. Included in the study were children and adolescents, along with their parents, who were asked to complete the questionnaires. If the parents were divorced and the child alternated between homes, the parent who shared residence with the child most of the time was asked to participate. Participants (parents and children) who did not complete the second questionnaire at the end of rehabilitation were still included in the study.

### 2.4 Ethics

The study was registered and approved by the Department of Research and Legacy in the Capital Region of Denmark. Ethical approval was not required according to Danish national legislation, as the study only involved questionnaire data. Written consent for the study was obtained from participants of legal age or their parents, in accordance with applicable regulations.

### 2.5 Questionnaire

PedsQL™ MFS is an 18-item questionnaire designed as a generic symptom-specific instrument to measure fatigue in pediatric patients (29). The PedsQL MFS provides parallel child self-reports and parent proxy reports. For both report types, the Danish versions of the questionnaire were administered, covering Toddler (2–4), Young Child (5–7), Child (8–12), Adolescent (13–18), and Young Adult (18–25). The survey provides a Total Score and three subscale scores (6 items in each subscale): General Fatigue (e.g., “I feel tired” and “I feel too tired to do things that I like to do”), Sleep/Rest Fatigue (e.g., “I feel tired when I wake up in the morning” and “I rest a lot”), and Cognitive Fatigue (e.g., “It is hard for me to keep my attention on things” and “It is hard for me to think quickly”). The English version of the questionnaire is validated for both children and young adults (29, 37) and has demonstrated good psychometric properties (38).

The items are scored on a five-point Likert scale and ask how often the respondent experienced a specific problem during the last month. Responses are converted using the PedsQL scoring key and a formula that translates the scores from 0 to 4 into a 0–100 scale (0 = 100, 1 = 75, 2 = 50, 3 = 25, 4 = 0). Higher scores indicate less fatigue, while lower scores indicate more severe fatigue (39).

### 2.6 Statistical analysis

Descriptive demographic and health information is presented for the full sample. For participants with both child/adolescent and parent ratings available, differences between mean scores were evaluated using paired *t*-tests. To evaluate the effects of the cause of injury, five subgroups were defined, and the mean score of each subgroup was compared to the overall mean of the five subgroups. The statistical significance of changes in PedsQL scores between the first and second assessments was tested using a linear mixed model. To account for the correlation between the two repeated measures, this model included participant as a random factor and time (first or second assessment) as a fixed effect. All analyses have been adjusted for participant gender and time since injury. The latter variable was never significant, while female gender was significant for child/adolescent rating of “Sleep/Rest Fatigue” and parent rating of “General Fatigue”.

## 3 Results

### 3.1 Characteristics of participants

Table 1 provides an overview of the demographic characteristics, injury types, and assessment intervals of the study participants. In total, 38 children or adolescents (21 boys and 17 girls) and/or their parents participated. The different injury types included “Stroke” (*n* = 15), “Tumor” (*n* = 8), “Infection” (*n* = 6), “TBI,” (*n* = 5) and “Other” (*N* = 4). A total of 103 reports were collected (49 self-reports from children and 54 parent proxy reports). On average, the first assessment was conducted 16.19 months after the brain injury occurred, with an average of 6 months (179.85 d) between the first and second assessments Table 1.

**Table 1:** Characteristics of participants.

	Total	1. Assessment	2. Assessment
N	38		
Sex (M)	21 (55,3)		
Age (years)		11.82 (4.47)	
Adolescents ≥13	20 (52,6)		
Type of injury			
Stroke	15 (39,5)		
Tumor	8 (21,0)		
Infection	6 (15,8)		
TBI	5 (13,2)		
Other^a^	4 (10,5)		
Interval (days between assessments)			179.85 (56.10)
Time since injury (months)		16.19 (34.37)	
Reports			
Child/self report	49	29	20
Parent report	54	34	20

^a^ Hydrocephalus (1), Epilepsy (2), Surgery (temporal lobe epilepsy) (1).

### 3.2 Self-reported and parent-reported fatigue—first and second. assessment

Table 2 displays the PedsQL MFS total scores and domain scores for children/adolescents (self-reports) and their parents at the first and second assessments. Parent ratings were generally lower than the self-reported scores from children/adolescents, indicating that parents perceived higher levels of fatigue. In the first assessment, the lowest mean score reported by parents was in the “Cognitive Fatigue” domain (46.32), while the highest mean score was in the “Sleep/Rest Fatigue” domain (57.23), indicating that parents observed the most fatigue-related symptoms in cognition and the least in sleep. This pattern persisted in the second assessment Table 2.

**Table 2:** Self-reported and parent reported fatigue—1. and 2. assessment.

	PedsQL scores first and second assessment			
	1. Assessment	2. Assessment	Cohen's d	Mixed model
N	29–34	20		
Child ratings	Mean (SD)	Mean (SD)		
General	65.23 (15.72)	70.21 (16.07)	0.31	0.041*
Sleep	55.51 (16.04)	62.71 (18.11)	0.43	0.020*
Cognition	62.93 (22.50)	67.92 (20.28)	0.23	0.329
Total	61.66 (15.10)	66.95 (16.43)	0.34	0.005**
Parent ratings	Mean (SD)	Mean (SD)		
General	52.82 (21.49)	55.63 (19.32)	0.14	0.435
Sleep	57.23 (22.67)	62.29 (19.98)	0.23	0.444
Cognition	46.32 (22.23)	52.92 (18.79)	0.31	0.259
Total	52.12 (19.73)	56.94 (17.32)	0.26	0.265

* *p* < 0.05.

** *p* < 0.01.

In contrast, the pattern for children/adolescents differed from that of the parents. At the first assessment, the lowest score, indicating the most fatigue, was in the “Sleep/Rest Fatigue” domain (55.51), while the highest mean score was in the “General Fatigue” domain (65.23). This pattern also persisted at the second assessment.

The PedsQL MFS total mean scores were 61.66 for the children/adolescents and 52.12 for the parent ratings. Paired *t*-tests for participants with both child/adolescent and parent ratings revealed significant differences between the two ratings across all subscales, except for the “Sleep/Rest Fatigue” scale.

#### 3.2.1 Fatigue development between the first and second assessment

Table 2 also presents the mean scores for both the first and second assessments, along with Cohen's d for the changes between assessments and the corresponding *p*-values based on a mixed model. Across all ratings, the second assessment showed higher mean scores (indicating less fatigue) than the first assessment. However, a significant change was observed in only three PedsQL ratings for the children/adolescents’ group: “General Fatigue,” “Sleep/Rest Fatigue,” and “Total Score”. For parent ratings, the improvement was not statistically significant.

The data indicate a modest degree of change over time, with effect sizes (Cohen's d) being slightly larger in the children/adolescents’ ratings than in the parents’ ratings. All effect sizes were <0.50, which is considered small according to Cohen's guidelines (40, 41). Further research is needed to determine how changes in domains such as sleep quality translate into meaningful improvements in daily life for children with ABI.

### 3.3 Differences and correlations between children and parent ratings (paired *t*-test)

Table 3 presents the results of a paired *t*-test and correlation analysis comparing children's and parents’ ratings of the child's fatigue in various domains during the first and second assessments. At the first assessment, 25 participants with both child/adolescent and parent ratings were available, while 15 ratings were available at the second assessment. In both assessments, the correlations for “Sleep,” “Cognitive Fatigue,” and “Total Score” were significant and consistently strong between the children's and parents’ ratings. In contrast, the “General Fatigue” domain showed a smaller, non-significant correlation Table 3.

**Table 3:** Differences and correlations between children and parent ratings (paired *t*-test).

1. Assessment (*n* = *25*)							
Domain	Children	Parents	Dif.	Dif./SD_pooled_	*t*.	*P* value	Correlation
General	64.83 (16.93)	53.33 (21.55)	11.50	0.65	2.46	0.022	0.28
Sleep	54.17 (16.45)	56.67 (21.52)	−2,50	−0,09	−0,76	0.045	0.66
Cognition	63.17 (22.65)	49.33 (22.14)	13.83	0.74	3.55	0.002	0.62
Total	60.72 (15.71)	53.11 (19.35)	7.61	0.54	2.51	0.019	0.64
2. Assessment (*n* = *15*)							
Domain	Children	Parents	Dif.	Dif./SD_pooled_	*t*.	*P* value	Correlation
General	69.72 (17.21)	56.94 (20.81)	12.78	0.54	2.16	0.049	0.29
Sleep	64.72 (18.49)	61.67 (20.36)	03.06	0.02	0.67	0.513	0.59
Cognition	71.11 (19.19)	52.22 (18.02)	18.89	0.77	4.09	0.001	0.54
Total	68.52 (16.76)	56.94 (17.48)	11.58	0.59	3.11	0.008	0.65

### 3.4 Analyses of causes of injury (TBI, stroke, infection, tumors, and other causes)

#### 3.4.1 Cause of injury

Table 4 provides an analysis of the first assessment ratings based on five different causes of injury. The sample was divided into five groups according to the injury types shown in Table 1, as follows: TBI, stroke, infection, tumors, and other causes. The coefficients represent the differences between the mean of each subgroup and the overall mean of the five subgroups. Table 4A shows the results for children/adolescents ratings. For the “Cognitive Fatigue” and “Total Score” subscales of the PedsQL, the coefficients indicate significantly higher mean scores for the stroke subgroup and significantly lower mean scores for the infection subgroup, suggesting that stroke is associated with less fatigue and infection with more fatigue. For “General Fatigue” and “Sleep/Rest Fatigue,” the coefficients followed a similar but non-significant trend. Table 4B displays the parent ratings, which showed a similar pattern: the stroke subgroup had significantly higher scores on the “General Fatigue,” “Cognitive Fatigue,” and “Total Score” scales, while the infection subgroup had non-significantly lower scores across all four PedsQL subscale”) while the infection subgroup obtained non-significant lower scores on all four PedsQL ratings Table 4.

**Table 4:** Analyses of causes of injury (TBI, stroke, infection, tumors, and other causes).

A. Child PedsQL ratings								
	General		Sleep		Cognition		Total	
Variable	Coefficient	*P* value	Coefficient	*P* value	Coefficient	*P* value	Coefficient	*P* value
Stroke	6.820	0.149	9.166	0.064	16.375	0.009**	9.907	0.020*
Infection	−8.320	0.182	−8.471	0.185	−21.125	0.011*	−13.519	0.017*
Tumor	0.012	0.998	7.359	0.248	10.543	0.181	5.093	0.340
Trauma	10.640	0.120	0.141	0.985	−1.333	0.874	6.668	0.306
Other	−9.153	0.228	−8.196	0.289	−4.459	0.638	−8.149	0.213
B. Parental PedsQL ratings								
	General		Sleep		Cognition		Total	
Variable	Coefficient	*P* value	Coefficient	*P* value	Coefficient	*P* value	Coefficient	*P* value
Stroke	13.362	0.027*	9.306	0.154	14.103	0.024*	12.258	0.025*
Infection	−9.553	0.207	−13.450	0.111	−13.087	0.096	−12.029	0.083
Tumor	8.005	0.262	5.002	0.523	4.671	0.521	5.893	0.360
Trauma	0.865	0.921	6.341	0.513	4.969	0.581	4.058	0.609
Other	−12.680	0.154	−7.199	0.458	−10.656	0.241	−10.180	0.204

* *p* < 0.05.

**p < 0.01.

Defined by the cause of injury, and for each subgroup the coefficient represents the difference between the mean of the group and the overall mean of the five groups. “*P* value” refers to a test of whether the individual mean deviates significantly from the overall mean.

## 4 Discussion

This study was conducted to investigate how children/adolescents and their parents perceive fatigue symptoms at the beginning and end of intensive rehabilitation. Additionally, we aimed to explore whether the etiology of the injury influences the nature of fatigue.

### 4.1 Experiences of fatigue during rehabilitation

The results in Table 2 indicate that parents generally scored lower on the PedsQL-MFS than children and adolescents did in self-assessments. This suggests that parents perceive their children as experiencing a higher degree of fatigue than the children and adolescents report themselves. Significant differences were observed between parent and child ratings on all scales except for the sleep-fatigue scale. Similar discrepancies in symptom perception between parents and children have been documented in other pediatric diagnostic areas (42, 43), as well as in studies within the pediatric ABI domain (10, 44, 45). These findings underscore the importance of obtaining both child/adolescent and parent perspectives when designing family-centered interventions for managing fatigue. Additionally, the results showed that the lowest scores (indicating the most severe symptoms) were on the parental ratings for the Cognitive fatigue subscale. This finding is consistent with previous research (10, 11, 42). Several factors may explain why children and adolescents do not report symptoms in this domain to the same extent as their parents. First, cognitive impairments following ABI may hinder the child's ability to accurately assess their own cognitive abilities. Second, adults are often more attuned to the compensatory strategies and adaptive frameworks that the child employs in daily life, which the child may not fully recognize due to impaired cognition.

The results listed in Table 2 show that that both children/adolescents and parents reported a general improvement in fatigue symptoms between the two assessments. The increases in scores were significant for the children's ratings of fatigue in the “General Fatigue” and “Sleep/Rest Fatigue” subscales. To our knowledge, only a few studies have examined the development of fatigue symptoms in the pediatric ABI field. Allonsius et al. (46) found significant improvement in fatigue symptoms during the first year of outpatient rehabilitation for adolescents with ABI, reaching a plateau after two years. The most pronounced improvement was observed in “General Fatigue”. Conversely, a follow-up study by Chricton et al. (14), which examined fatigue after traumatic brain injury in childhood at 6- and 12-months postinjury, found that children and adolescents did not experience improvement but instead showed a deterioration in cognitive fatigue over time. In the adult population, varied patterns of fatigue recovery have been observed after traumatic brain injury (26, 47), with some studies indicating improvement, some showing deterioration, and others reporting sustained reduced levels of fatigue over time. The results of our study may be attributed to several factors. Some children and adolescents may be severely affected both physically and mentally at the beginning of rehabilitation but experience spontaneous recovery over time due to gradual neurological improvement, better management of pain and anxiety, and other factors that could influence fatigue symptoms. The rehabilitation process itself, which includes both physical and cognitive training, may also contribute to the improvement observed from the first to the second assessment. Studies have shown that physical exercise, for instance, reduces fatigue symptoms in adolescents with cerebral palsy (48) and in children undergoing cancer treatment (49).

The improvement in symptoms was not as pronounced in parental reports as in the self-reports of children and adolescents. In our clinical practice, we observe that parents’ detailed knowledge of their children's daily activities and well-being decreases once the child transitions from hospitalization to rehabilitation, where parents no longer participate as closely in daily activities. We hypothesize that this affects the parents’ ability to accurately assess their children's fatigue during rehabilitation, as they primarily interact with their children in the afternoons and evenings after a full day of training. It is possible that children may notice small improvements in their fatigue symptoms before their parents do. However, further research is needed to confirm or refute this hypothesis.

### 4.2 Experiences of fatigue in subgroups (cause of injury)

The results listed in Table 4 show differences in the experience of fatigue at the beginning of rehabilitation based on various injury types. Children and adolescents with stroke reported significantly less cognitive fatigue and overall fatigue compared to the entire sample (including stroke) (Table 4A). In contrast, children and adolescents with ABI due to infection experienced significantly more symptoms on the same scales. Parental responses illustrated in Table 4B indicated that the stroke group scored significantly higher relative to the entire sample, showing less fatigue on all scales except for the sleep/rest fatigue scale. Conversely, the infection group had the lowest scores, corresponding to the most severe symptoms on all scales, although these results were not statistically significant. These findings should be interpreted with caution due to the small number of participants in the subgroups. Currently, few studies definitively determine whether our findings reflect real differences between subgroups or are coincidental. A study by Hypher et al. (50) investigated fatigue through parental responses to the PedsQL-MFS in a cohort of 127 children with ABI at least one year after injury. The study did not find a significant association between etiology and the degree of fatigue. However, children with brain tumors and infection reported the most fatigue symptoms, whereas children with stroke reported the second-fewest. The group with the fewest symptoms was the anoxic injury group, which was not represented in our study. Similarly, Wilkinson et al. (19), in their review comparing fatigue across different subgroups, found that children and adolescents who survived meningitis exhibited slightly higher levels of fatigue than other ABI groups, consistent with findings by Sumpter et al. (21). Additionally, a study comparing children and adolescents with TBI to those with ABI from other causes (such as brain tumors and infections affecting the brain and meninges) found that in parental ratings, the non-TBI group showed significantly more symptoms on the cognitive fatigue and total fatigue scales than the TBI group (10).

Research in the field consistently shows that all subgroups of children and adolescents with ABI experience significantly more fatigue than healthy control groups. However, our study, along with previous research, suggests that some subgroups may be at greater risk of experiencing fatigue symptoms than others, though current research cannot clearly identify which groups are most affected or how symptom patterns differ between groups. These differences may have important implications for planning assessments and treatment, with particular attention directed toward identifying children and adolescents at the highest risk of fatigue early in the rehabilitation process. The limited research in this area underscores the need for further, larger studies to explore the differences in fatigue presentation among various subgroups of pediatric ABI. This is clinically relevant because children and adolescents with different causes of injury are often treated together in rehabilitation centers, and assessment and intervention methods are typically designed for the entire population rather than tailored to specific injury subgroups.

### 4.3 Study strengths and limitations

The strength of this study lies in its longitudinal design. By collecting data at both the beginning and end of the rehabilitation period, the study provides valuable insights into the development of fatigue over time in children and adolescents with ABI. Additionally, the use of the Pediatric Quality of Life Inventory Multidimensional-Fatigue Scale (PedsQL MFS) allows for a comprehensive assessment of fatigue, encompassing general, sleep, and cognitive dimensions. By incorporating both self-reported data from children/adolescents and parental reports, the study highlights the importance of considering multiple perspectives in the evaluation of fatigue. The inclusion of participants with varying types of ABI, including traumatic brain injury, stroke, infection, tumors, and other causes, enriches our understanding of how fatigue manifests across different etiologies. Finally, the study's focus on children and adolescents undergoing interdisciplinary neurorehabilitation has practical implications, potentially guiding clinical interventions tailored to the unique needs of this population.

However, the study also has several limitations. First, the relatively small sample size limits the generalizability of the findings. The cohort is also highly heterogeneous, with participants differing in age, cause, and severity of injury, as well as time since injury to the first assessment. While the inclusion of diverse etiologies is a strength, it also complicates drawing specific conclusions for each subgroup, given the small number of participants in each category. Another limitation is the reliance on self-reported measures of fatigue, which may introduce bias or reduce the accuracy of the data, as perceptions can vary over time or be influenced by subjective factors. Incorporating objective measures, such as physiological monitoring, would enhance future studies by providing a more comprehensive understanding of the relationship between fatigue and activity levels.

Moreover, the study only provides insights into short-term changes in fatigue during rehabilitation. Long-term follow-up assessments would offer a deeper understanding of fatigue trajectories, which is essential given the lack of knowledge about the long-term development of fatigue in this population. Lastly, a notable limitation is the absence of a comparison to a Danish norm group, preventing a comparison of the study participants to a healthy population of children and adolescents.

## 5 Conclusion

This study has contributed to the understanding of fatigue in children and adolescents with ABI through several key findings. It highlights a disparity in the perception of fatigue between children/adolescents and their parents, with parents consistently assessing their children's fatigue as more pronounced than the children themselves report. This underscores the importance of a comprehensive approach that integrates both perspectives to achieve a more nuanced and holistic understanding of fatigue. The results also suggest a general improvement in fatigue levels from the beginning to the end of the rehabilitation period. Both children/adolescents and parents reported positive developments, particularly in overall well-being and sleep. These findings may indicate that intensive rehabilitation, among other factors, has a beneficial impact on fatigue levels in this population.

Furthermore, the study observed differences in fatigue experiences based on the cause of brain injury. Children and adolescents with stroke appeared to experience less fatigue, while those with infections reported more pronounced fatigue. These observations emphasize the need for differentiated intervention strategies tailored to the specific etiology of the brain injury.

Our hope is that this study will stimulate further research into fatigue. Increased knowledge in this area will benefit the vulnerable population of children with pediatric acquired brain injury and could also extend to children with congenital brain damage and/or cerebral palsy, who often experience mental fatigue (45).

## Data Availability

The raw data supporting the conclusions of this article will be made available by the authors, without undue reservation.
